# Clinical Characteristics and Perioperative Complication Profiles of COVID-19–Positive Patients Undergoing Hip Fracture Surgery

**DOI:** 10.5435/JAAOSGlobal-D-21-00104

**Published:** 2021-10-15

**Authors:** Anoop R. Galivanche, Michael R. Mercier, Christopher A. Schneble, Jordan Brand, Neil Pathak, Arya G. Varthi, Lee E. Rubin, Jonathan N. Grauer

**Affiliations:** From the Department of Orthopaedics and Rehabilitation, Yale School of Medicine, New Haven, CT.

## Abstract

**Introduction:**

The coronavirus 2019 (COVID-19) pandemic disease has imposed an unprecedented degree of stress on healthcare systems. This study aimed to understand whether COVID-19 positivity is associated with an increased risk of adverse outcomes after geriatric hip fracture surgery.

**Methods:**

From a national administrative claims data set, patients who underwent hip fracture surgery from April 1, 2020, to December 1, 2020 were selected for analysis. COVID-19–positive status was assessed by the emergency International Classification of Diagnoses, 10th Revision, COVID-19 code within 2 weeks before the surgery. Demographic, comorbidity, and 30-day postoperative adverse event information were extracted. Logistic regression before and after 10:1 propensity matching was performed to identify patient risk factors associated with the occurrence of postoperative adverse events.

**Results:**

Of 42,002 patients who underwent hip fracture surgery, 678 (1.61%) were identified to be positive for COVID-19 infection. No significant differences in age, sex, and procedure type were found between COVID-19–positive and COVID-19–negative groups, but the COVID-19–positive patients demonstrated a higher incidence of several comorbidities. These differences were no longer significant after matching. After matching, the COVID-19–positive group had a higher incidence of any, serious, and minor adverse events (*P* < 0.001 for all). Controlling for preoperative variables, COVID-19 positivity was associated with an increased risk of experiencing any adverse events (odds ratio [OR] = 1.62, 95% confidence interval [95% CI] = [1.37 to 1.92], *P* < 0.001), serious adverse events (OR = 1.66, 95% CI = [1.31 to 2.07], *P* < 0.001), and minor adverse events (OR = 1.59, 95% CI = [1.34 to 1.89], *P* < 0.001).

**Discussion:**

After matching and controlling for confounding variables, COVID-19–positive hip fracture patients had increased odds of multiple postoperative events. Clinicians caring for this vulnerable geriatric population should be mindful of this risk to improve the care for these patients during the ongoing global pandemic.

The coronavirus 2019 (COVID-19) pandemic disease has imposed unprecedented stress on healthcare systems worldwide.^1^ To mitigate the risk of viral transmission and preserve hospital resources, suspensions of all but urgent and emergent surgeries had been intermittently implemented throughout the pandemic in the United States.^2,3^ Hip fractures are a common pathology that require urgent stabilization in a timely fashion to facilitate mobilization and limit secondary complications and mortality. As such, hip fracture patients were triaged to undergo surgery regardless of COVID-19 status. To date, whether COVID-19–positive status is associated with increased complications in this population remains unknown.

Hip fracture management has been an important target of optimization within orthopaedic surgery, given the aging population and the high expenses associated with hip fracture treatment. A 2015 analysis estimated the mean annual incidence of hip fractures as 957.3 per 100,000 in the United States.^4^ Much work has been done to develop guiding principles for the management of hip fractures,^5^ such as prioritizing the timing of surgery, because treatment of these fractures within 48 hours has been shown to reduce complications.^6^

Unfortunately, the elderly patients who tend to present with hip fractures have continued to comprise a substantial portion of the COVID-19 burden in the United States, according to surveillance reports by the Centers for Disease Control.^7,8^ As such, it is unavoidable that patients with hip fractures will also present with COVID-19 although no epidemiologic studies have formally quantified the coincidence of hip fractures and COVID-19 positivity.

COVID-19–positive patients not only pose a potential risk to operating room personnel if needing surgery but also are at increased risk for postoperative complications.^9,10^ A multicenter study at 235 hospitals in 24 countries found higher rates of complications within 30 days of index surgical procedure in COVID-19–positive patients than those in COVID-19–negative patients. Notably, patients were deemed positive for COVID-19 infection even if they tested positive for COVID-19 after their procedure. However, this study was not limited to orthopaedic surgery patients, nor did it report specifically on hip fracture surgery patients.

Evidence-based guidance for managing COVID-19–positive hip fracture patients during the perioperative period has been sparse, with much of the existing literature based on expert consensus and opinion.^11-13^ Mi et al^14^ reported on the clinical courses of 10 fracture patients who were positive for COVID-19 infection, finding that COVID-19–positive patients had more severe clinical courses than would be expected of COVID-19–negative fracture patients.

There remains a need to better understand the effect of COVID-19 on surgical outcomes. This study sought to use real-world data to describe the clinical characteristics, comorbidity, and postoperative complication profiles of COVID-19–positive patients who underwent hip fracture surgery in the United States during the pandemic, with the hypothesis that COVID-19 positivity was associated with greater risk of adverse events after hip fracture surgery.

## Methods

### Data Source and Patient Population

This study used the Symphony Healthcare subset of the Datavant COVID-19 Research Database. The Symphony subset includes data on more than 280 million patients, 1.8 million prescribers, and 16,000 health plans in the United States. Symphony data set was requested from the Datavant consortium by the submission of a hypothesis-driven application. This application was then reviewed by the consortium's Scientific Steering Committee, Patient Advocacy and Ethics Advisors, and Privacy Advisors. After review, the application for data access was approved. Our institution's Human Investigations Committee exempted this study from further review.

The data warehouse was queried for diagnoses of hip fracture using International Classification of Diagnoses, 10th Revision (ICD-10), diagnostic codes M84.X and S72.X. To identify instances of hip fracture surgical fixation, the data warehouse was queried for the Current Procedural Terminology codes: 27235, 27236, 27244, and 27245 (Table 1). The two data frames were then intersected, to yield a list of insurance claims representing instances of surgical treatment of hip fractures.

**Table 1:** Demographic Characteristics of Patients by COVID-19 Diagnosis

Total patients = 42,002	COVID-19 (−)		COVID-19 (+)		^a^ *P*	10:1 Matched COVID-19 (−) Cohort		^a^ *P*
	Number	Percent	Number	Percent		Number	Percent	
	41,324	98.39	678	1.61		6780	50.00	
Age					0.089			0.437
60-69.9	6320	15.29	87	12.83		760	11.21	
70-79.9	11,789	28.53	214	31.56		2154	31.77	
80+	23,215	56.18	377	55.60		3866	57.02	
Sex (n = 42,001)					0.898			0.615
Men	12,834	31.06	216	31.86		2091	30.84	
Women	28,489	68.94	462	68.14		4689	69.16	
Procedure type					0.187			0.895
Percutaneous pinning	3248	7.86	47	6.93		427	6.30	
Open treatment	15,112	36.57	245	36.14		2411	35.56	
IM plate/screw	1297	3.14	13	1.92		131	1.93	
IM nail	21,667	52.43	373	55.01		3811	56.21	

COVID = coronavirus 2019 disease

a Statistically significant at *P* < 0.05.

Propensity score matched on the basis of age, sex, procedure type, and medical comorbidities.

This list of insurance claims was then deduplicated using unique patient identifiers available in the data warehouse and distinct dates of insurance claims. In this manner, a unique list of hip fracture patients undergoing surgical intervention was generated.

Patients who underwent surgery from April 1, 2020, through December 1, 2020, were identified. Patients with the above-noted codes who were aged at least 60 years and had 30-days of follow-up were included in the study. Patients were classified as positive for COVID-19 if they had an insurance claim with the ICD-10 diagnosis code U07.1 within two weeks before the date of surgery. Patients are assigned this emergency ICD-10 code if they have a laboratory-confirmed diagnosis of COVID-19.

Patient age, sex, income bracket, and partial zip code were extracted from the data warehouse for each instance of hip fracture surgery. The presence of the following comorbidities was assessed for each patient: asthma, hypertension, chronic kidney disease, congestive heart failure, chronic obstructive pulmonary disease, coronary artery disease, diabetes mellitus, obesity, and tobacco use disorder. A list of ICD-10 codes used to identify comorbidities is provided in Appendix 1, http://links.lww.com/JG9/A164.

### Postoperative Adverse Events

Hip fracture surgery patients were then queried for the presence of serious adverse events (SAEs), minor adverse events (MAEs), and any adverse events (AAEs) within 30 days of index procedure. This was done based on ICD-10 diagnostic codes, as listed in Appendix 2, http://links.lww.com/JG9/A164.

SAE was defined by the occurrence of at least one of the following complications: surgical site infection, sepsis, venous thromboembolism (VTE) events, cardiac arrest, acute myocardial infarction, and pancreatitis. MAE was defined by the occurrence of at least one of the following complications: pneumonia, urinary tract infection, acute kidney injury, and wound dehiscence. AAE was defined by the occurrence of an MAE or SAE. Definitions of SAE and MAE were based on previously reported classifications.^15^

### Data Analysis

Univariate analyses were used to compare patient demographic and comorbidity variables using Pearson's chi-squared tests with Yates continuity correction. Incidences of complications were also compared using Pearson chi-squared tests with Yates continuity correction.

Multiple logistic regression was then performed. After generation of surgical complication bins, generalized linear models with binomial family distributions and logit link functions were constructed, using demographic, comorbidity, and COVID-19 diagnosis variables as covariates. Odds ratios (ORs) for each adverse event type were calculated. VTE and incidence of pneumonia were evaluated as separate end points. The COVID-19–negative cohort from the same database was used as the referent.

Propensity score matching was then performed to adjust for imbalances in comorbidity burden between the COVID-19–positive and COVID-19–negative cohorts. Each COVID-19–positive patient was matched to 10 COVID-19–negative patient on the basis of demographic and comorbidity variables. Generalized linear models were again constructed to derive odds of experiencing adverse events, with the COVID-19–negative cohort as the referent.

Aggregate insurance claim data were hosted on a Snowflake data warehouse (Snowflake), accessible by a secure remote Amazon Workspace instance (Amazon.com). All statistical analyses were performed using R (R Foundation for Statistical Computing) with computing packages dplyr (dplyr: A Grammar of Data Manipulation. R package version 0.8.5), magrittr (magrittr: A Forward-Pipe Operator for R. R package version 1.5), and MatchIt (MatchIt: Nonparametric Preprocessing for Parametric Causal Inference). The alpha level for statistical significance was set at 0.05 for all tests.

## Results

### Patient Population

In total, 42,002 patients met criteria for inclusion in the study. Of these, 678 (1.61%) had tested positive for COVID-19 in the 2 weeks preceding their hip fracture surgery (Table 1). No statistically significant differences in age, sex, or hip fracture procedure type were observed between COVID-19–positive and COVID-19–negative patients.

Comorbidity characteristics were compared between the two cohorts (Table 2). On univariate chi-squared analysis, COVID-19–positive patients were more likely to present with asthma (8.11% compared with 5.99%, *P* = 0.027), chronic kidney disease (32.60% compared with 25.00%, *P* < 0.001), congestive heart failure (30.83% compared with 22.79%, *P* < 0.001), chronic obstructive pulmonary disease (26.40% compared with 21.74, *P* = 0.004), coronary artery disease (32.89% compared with 28.00%, *P* = 0.006), diabetes (35.84% compared with 27.23%, *P* = 0.008), hypertension (82.45% compared with 70.60%, *P* = 0.001), and obesity (12.54% compared with 9.36%, *P* = 0.006).

**Table 2:** Comorbidities of Patients by COVID-19 Diagnosis

Total patients = 42,002	COVID-19 (−)		COVID-19 (+)		^a^ *P*	10:1 Matched COVID-19 (−) Cohort		^a^ *P*
	Number	Percent	Number	Percent		Number	Percent	
	41,324	98.39	678	1.61		6780	50.00	
Asthma	2477	5.99	55	8.11	**0.027**	498	7.35	0.516
Chronic kidney disease	10,329	25.00	221	32.60	**<0.001**	2161	31.87	0.733
Congestive heart failure	9416	22.79	209	30.83	**<0.001**	2057	30.34	0.827
COPD	8982	21.74	179	26.40	**0.004**	1718	25.34	0.576
Coronary artery disease	11,570	28.00	223	32.89	**0.006**	2146	31.65	0.537
Diabetes	11,253	27.23	243	35.84	**<0.001**	2364	34.87	0.642
Hypertension	29,176	70.60	559	82.45	**<0.001**	5592	82.48	1.000
Obesity	3869	9.36	85	12.54	**0.006**	846	12.48	1.000
Tobacco use	4723	11.43	70	10.32	0.403	598	8.82	0.216

a COVID-19 = Coronavirus 2019 diseaseStatistically significant at *P* < 0.05.

Propensity score matched on the basis of age, sex, procedure type, and medical comorbidities. Bolding indicates statistical significance at p<0.05.

A 1:10 COVID-19–negative cohort was constructed and propensity matched on the basis of age, sex, procedure type, and comorbidities for comparison with the COVID-19–positive cohort. The propensity-matched cohort's demographic data are summarized in Table 1 and comorbidity data are in Table 2. After propensity score matching, no differences in these variables were found between the COVID-19–positive and COVID-19–negative cohorts.

### Postoperative Adverse Events

Adverse events occurring within the 30-day postoperative period were then compared between the two cohorts (Table 3). COVID-19–positive patients were more likely to experience AAEs (40.12% compared with 25.46%, *P* < 0.001), SAEs (15.49% compared with 8.27%, *P* < 0.001), and MAEs (34.51% compared with 21.58%, *P* < 0.001).

**Table 3:** Adverse Events by COVID-19 Diagnosis

Complication	COVID-19 (−)		COVID-19 (+)		^a^ *P*
Total patients = 42,002	41,324	98.39	678	1.61	
AAE	10,523	25.46	272	40.12	**<0.001**
SAE	3419	8.27	105	15.49	**<0.001**
Surgical site infection	172	0.42	1	0.15	
Sepsis	1127	2.73	47	6.93	
Venous thromboembolism	1417	3.43	45	6.64	**0.002**
Cardiac arrest	254	0.61	7	1.03	
MI	832	2.01	22	3.24	
Pancreatitis	42	0.10	0	0.00	
MAE	8916	21.58	234	34.51	**<0.001**
Pneumonia	1883	4.56	76	11.21	**<0.001**
UTI	4295	10.39	99	14.60	
Acute kidney injury	4293	10.39	107	15.78	
Wound dehiscence	208	0.50	5	0.74	

AAE = any adverse event, COVID-19 = Coronavirus 2019 disease, MAE = minor adverse event, SAE = serious adverse event, MI = myocardial infarction, UTI = urinary tract infection

a Bolding indicates statistical significance at *P* < 0.05

Regarding individual adverse events, COVID-19–positive patients had higher rates of the following complications: sepsis (6.93% compared with 2.73%), a VTE event (6.64% compared with 3.43%), cardiac arrest (1.03% compared with 0.61%), myocardial infarction (3.24% compared with 2.01%), pneumonia (11.21% compared with 4.56%), urinary tract infection (14.60% compared with 10.39%), acute kidney injury (15.78% compared with 10.39%), and wound dehiscence (0.74% compared with 0.50%). These findings are summarized in Table 3.

A multiple regression model controlling for age, sex, comorbidity burden, and procedure type was constructed to determine the odds of postoperative adverse event occurrence. This approach allowed for the quantification of risk for adverse events in the context of several demographic and preoperative factors. For the aggregated adverse event categories, increased odds of AAEs (OR = 1.70; 95% confidence interval [CI], 1.44 to 2.00; *P* < 0.001), SAEs (OR = 1.78; 95% CI, 1.43 to 2.20; *P* < 0.001), and MAEs (OR = 1.65; 95% CI, 1.39 to 1.95; *P* < 0.001) were found in COVID-19–positive patients.

**Figure 1:**
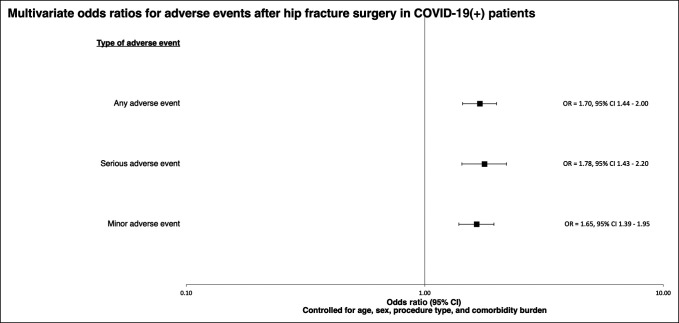
A forest plot showing the odds ratios and associated 95% confidence intervals for postoperative adverse events in COVID-19–positive hip fracture patients, using the aggregate COVID-19–negative hip fracture cohort as the referent. The logistic regression models generating these odds ratios controlled for age, sex, procedure type, and comorbidity burden. COVID-19 = Coronavirus 2019 disease

Separate models were constructed to evaluate ORs for VTE and pneumonia, given literature describing the association between COVID-19 and these complications. COVID-19–positive patients also had increased odds of VTE (OR = 2.06; 95% CI 1.81 to 2.44; *P* < 0.001) and pneumonia (OR = 2.31; 95% CI 1.78 to 2.95; *P* = 0.001). These findings are presented in Table 4 and Figure 1.

**Table 4:** Multivariate and Propensity Score–Matched ORs by COVID-19 Status

Complication	Multivariate OR		
	Controlled for Preoperative Variables^a^		
	Propensity–Matched Multivariate Odds Ratio^b^		
Total patients = 42,002	OR	95% CI	*P*
AAE	**1.70**	**1.44-2.00**	**<0.001**
	**1.62** ^b^	**1.37-1.92**	**<0.001**
SAE	**1.78**	**1.43-2.20**	**<0.001**
	**1.66** ^b^	**1.31-2.07**	**<0.001**
Venous thromboembolic event	**2.06**	**1.81-2.44**	**<0.001**
	**1.69** ^b^	**1.20-2.32**	**0.002**
MAE	**1.65**	**1.39-1.95**	**<0.001**
	**1.59** ^b^	**1.34-1.89**	**<0.001**
Pneumonia	**2.31**	**1.78-2.95**	**<0.001**
	**§ 2.16**	**1.64-2.81**	**<0.001**

AAE = any adverse event, CI = confidence interval; COVID = Coronavirus 2019 disease; MAE = minor adverse event, OR = odds ratio, SAE = serious adverse event

a Preoperative variables controlled for included age, sex, procedure type, and medical comorbidities.

b Propensity score matched on the basis of age, sex, procedure type, and medical comorbidities.

Bolding indicates statistical significance at *P* < 0.05.

**Figure 2:**
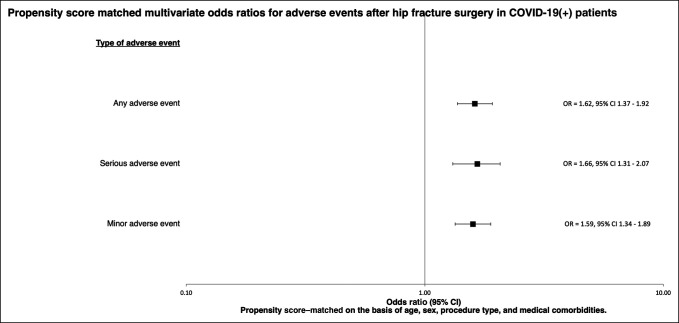
A forest plot showing the odds ratios and associated 95% confidence intervals for postoperative adverse events in COVID-19–positive hip fracture patients, using a 1:10 propensity score–matched COVID-19–negative hip fracture cohort as the referent. The referent COVID-19–negative hip fracture cohort was matched to COVID-19–positive cases based on age, sex, procedure type, and comorbidity burden. COVID-19 = Coronavirus 2019 disease

Similarly, multivariable regression models were constructed to determine the odds of adverse events using the propensity score–matched COVID-19–negative cohort as the referent. For the aggregated adverse event categories, significantly increased odds were noted for AAEs (OR = 1.62; 95% CI, 1.37 to 1.92; *P* < 0.001), SAEs (OR = 1.66; 95% CI, 1.31 to 2.07; *P* < 0.001), MAEs (OR = 1.59; 95% CI, 1.34 to 1.89; *P* < 0.001), VTE (OR = 1.69; 95% CI 1.20 to 2.32; *P* = 0.002), and pneumonia (OR = 2.16; 95% CI 1.64 to 2.81; *P* < 0.001). These findings are presented in Table 4 and Figure 2.

## Discussion

Patients undergoing geriatric hip fracture surgery are a relatively compromised population at baseline and at risk for adverse events.^4^ Given that COVID-19 has continued to affect geriatric patients, the compounded considerations inherent to this patient population and COVID-19 are not previously well characterized.^7,8^ To date, previous studies looking at this question were conducted in single institution samples, with limited statistical power.^14^

Drawing from a large, national insurance claims database tracking patients from April 1, 2020, to December 1, 2020, in the United States, this study compared the preoperative characteristics and postoperative adverse events of COVID-19–positive hip fracture patients with COVID-19–negative geriatric hip fracture patients. As hypothesized, COVID-19–positive geriatric hip fracture patients were found to be at significantly elevated odds of adverse events compared with COVID-19–negative patients, even after controlling for patient demographic factors and differential comorbidity burden.

The overall COVID-19 positivity rate for geriatric patients with hip fracture in this studied sample was 1.61%, as approximated by utilization of the emergency diagnostic code U07.1. National estimates of COVID-19 positivity have varied by patient demographics and geographic region. These estimates continue to change with testing availability and viral contagion.^16,17,18,19^ The emergency testing code U07.1 is only ascribed to COVID-19–positive patients confirmed by laboratory tests and was introduced in April 2020. Therefore, the positivity rate reported in this study is likely an underestimation of the true positivity rate of hip fracture patients. Physicians should be aware of potential COVID-19 positivity in patients and continue to test asymptomatic patients before surgical intervention per any existing guidelines.

In this study, COVID-19–positive patients had significantly greater comorbidity burdens than COVID-19–negative patients. This finding is reported in previous investigations of COVID-19–positive patients.^9,20-22^ In particular, COVID-19–positive patients had statistically significantly higher rates of chronic kidney disease, congestive heart failure, coronary artery disease, diabetes mellitus, and hypertension.

Without and with propensity matching, multivariable analyses found COVID-19–positive geriatric hip fracture patients to be at significantly greater odds of AAEs, SAEs, and MAEs within 30 days of index procedure. The findings of this study are concordant with previous work demonstrating the association of COVID-19 positivity with postoperative complication risk in an international cohort across multiple surgical specialties for indications pertaining to benign disease, cancer, trauma, and obstetrics.^9^

VTE is a specific adverse event that was found to be of greater odds for those who were positive for COVID-19 infection and bears specific discussion. Literature has reported a potential link between COVID-19 and coagulation abnormalities.^23,24,25,26^ In a previous study examining 3334 COVID-19–positive patients hospitalized in a large New York City health system, 16% of the patients experienced an arterial or venous thromboembolic event.^23^ Other studies found unique histologic hallmarks, suggesting a role for hypercoagulability in COVID-19 morbidity.^22,25,26^ In this study, both univariate and multivariable analyses corroborated the association between COVID-19 positivity with VTE events, although the exact timing of clinically significant VTE events remains uncertain. As a result, utilization of effective VTE prophylaxis is critical in this cohort.

COVID-19–positive patients also had a higher odds of pneumonia diagnosis after surgery, in both aggregate and propensity score–matched regressions. Pneumonia and other pulmonary complications were also found to occur at greater rates in the aforementioned international COVID-19 collaborative cohort. Previous research has proposed a putative molecular mechanism between severe acute respiratory syndrome coronavirus 2 (SARS-CoV-2) and pneumonia.^27^ In light of this and other research documenting the epidemiology of COVID and pneumonia, our finding is concordant and draws attention to its occurrence among orthopaedic surgery patients.^9^

This study does have several limitations within which the results should be interpreted. First, this study used the real-world evidence in the Symphony insurance claims data set, which is subject to the limitations inherent to administrative data sets.^28^ Nonetheless, administrative databases offer the ability to investigate questions for which prospective, randomized trials are unfeasible and single institution data inadequate. Because of this, the utilization of administrative databases has increased markedly over the past decade.^29^

In addition, the database does not reliably capture mortality as an end point because this leads to patients dropping out from being followed up by their insurance carriers. This limits our ability to examine the effect of COVID-19 positivity on mortality after hip fracture surgery. Next, our ability to identify COVID-19 positivity was predicated on the utilization of the emergency COVID-19 ICD-10 codes. The extent to which patients who are positive for COVID-19 infection are ultimately assigned the ICD-10 code when submitting insurance claims is unclear. Finally, patients who are positive for COVID-19 infection may have had more aggressive surveillance for complications such as VTEs and pneumonia, which could conceivably produce a detection bias in the COVID-19–positive cohort.

Overall, this study found that over 8 months in 2020, 1.6% of hip fracture surgery patients were positive for COVID-19 infection in the United States, and a significant association was found between COVID-19 positivity and postoperative adverse events. This association held after controlling for patient demographic factors and comorbidity burden in both multivariable regressions and propensity score–matched regressions for aggregated and specific adverse events. Given the medical complexity of managing hip fracture patients, the association between COVID-19 positivity with increased odds of adverse events, particularly pneumonia and VTE, may be used in preoperative counseling and developing postoperative treatment algorithms.
